# Combining a deep learning model with clinical data better predicts hepatocellular carcinoma behavior following surgery

**DOI:** 10.1016/j.jpi.2023.100360

**Published:** 2023-12-29

**Authors:** Benoit Schmauch, Sarah S. Elsoukkary, Amika Moro, Roma Raj, Chase J. Wehrle, Kazunari Sasaki, Julien Calderaro, Patrick Sin-Chan, Federico Aucejo, Daniel E. Roberts

**Affiliations:** a Owkin Lab, Owkin, Inc., New York, NY, USA; b Department of Pathology, Cleveland Clinic, Cleveland, OH, USA; c Department of Surgery, Cleveland Clinic, Cleveland, OH, USA; d Department of Surgery, Stanford University, Palo Alto, CA, USA; e Department of Pathology, Henri Mondor University Hospital, Créteil, France

**Keywords:** Hepatocellular carcinoma, Artificial intelligence, Machine learning, Deep learning, Prognostic modeling

## Abstract

Hepatocellular carcinoma (HCC) is among the most common cancers worldwide, and tumor recurrence following liver resection or transplantation is one of the highest contributors to mortality in HCC patients after surgery. Using artificial intelligence (AI), we developed an interdisciplinary model to predict HCC recurrence and patient survival following surgery. We collected whole-slide H&E images, clinical variables, and follow-up data from 300 patients with HCC who underwent transplant and 169 patients who underwent resection at the Cleveland Clinic. A deep learning model was trained to predict recurrence-free survival (RFS) and disease-specific survival (DSS) from the H&E-stained slides. Repeated cross-validation splits were used to compute robust C-index estimates, and the results were compared to those obtained by fitting a Cox proportional hazard model using only clinical variables. While the deep learning model alone was predictive of recurrence and survival among patients in both cohorts, integrating the clinical and histologic models significantly increased the C-index in each cohort. In every subgroup analyzed, we found that a combined clinical and deep learning model better predicted post-surgical outcome in HCC patients compared to either approach independently.

## Introduction

Hepatocellular carcinoma (HCC) represents the third leading cause of cancer-related death worldwide.^1^^,^^2^ Surgical treatment varies, with liver transplantation being the primary intervention for cirrhotic patients with early-stage HCC^3^^,^^4^ while surgical resection is the preferred treatment for patients with preserved underlying liver function. However, tumor recurrence following surgery represents one of the most common causes of death in these cohorts, with tumor recurrence seen in up to 20% of transplanted patients^4^, ^5^, ^6^, ^7^, ^8^ and up to 80% of patients who underwent resection.^9^, ^10^, ^11^

Various clinical and pathologic features have been shown to predict HCC recurrence and patient prognosis following liver resection or transplantation. These features include radiographic measurements of tumor size and number, serologic parameters such as alpha-fetoprotein (AFP) level, and pathologic features such as histologic grade and the presence or absence of vascular invasion.^12^, ^13^, ^14^ Our methods to stratify the risk of HCC recurrence have evolved over time, beginning with a landmark paper defining the Milan criteria, a system based solely on the pre-operative radiographic measurement of tumor extent, which has remained the benchmark for assessing transplant suitability in patients with HCC.^15^ Alternative prognostic models, such as HALT-HCC,^16^ have been proposed that are based exclusively on clinical variables such as AFP level. Purely histologic prognostic models have also been developed, such as the Recurrence Risk Assessment Score (RRAS).^17^ More recently, hybrid models have been developed that integrate variables from multiple disciplines to better predict HCC recurrence. The RETREAT score^18^ is one example, which combines both clinical and pathologic features. These stepwise advancements in HCC prognostication have enabled us to continuously improve post-surgical surveillance strategies and identify patients who would benefit from adjuvant therapy.

Artificial intelligence (AI) and machine learning represent the next step in modeling HCC outcome. AI is being increasingly utilized in modern practice,^19^, ^20^, ^21^ and computational approaches to prognostic modeling have shown tremendous promise. We describe in this study an AI approach that integrates clinical variables with a deep learning model to predict tumor behavior in HCC patients who underwent liver resection or transplantation.

## Material and methods

### Patients and samples

We retrospectively identified patients with HCC who underwent liver transplantation or surgical resection at the Cleveland Clinic during a 17-year period from 2002 to 2018. Demographics, underlying liver disease, history of locoregional therapy, pre-operative serum AFP level, and follow-up data were obtained from the electronic medical records. Pre-operative imaging was reviewed. The imaging study performed most recently prior to any intervention (either chemoembolization or surgery if no locoregional therapy was performed) was reviewed. If multiple imaging modalities were obtained, MRI was preferentially reviewed over CT scans. In the resection cohort, between 1 and 3 representative digital slides of H&E-stained sections were available for each HCC case (svs format, 40× magnification, 385 slides in total from 169 patients). In the transplant cohort, 1 digital slide of a H&E-stained section was available for each HCC case (svs format, 40× magnification, 300 slides in total from 300 patients). The use of patient samples from the Cleveland Clinic was approved by the institutional review board.

### Convolutional neural networks for predicting patient survival

We used a deep-learning algorithm called "SCHMOWDER",^21^ which was specifically designed for the processing of whole-slide images (WSI). SCHMOWDER automatically identifies very localized survival-related patterns on slides and calculates a risk score for each WSI analyzed in 3 successive steps: a pre-processing step, a tile-scoring step, and a prediction step. First, segmentation is performed using a UNet neural network to separate tissue from background on the WSI. The background is discarded and the tissue area is divided into small squares called “tiles” that are 112×112 micrometers in size (224 pixels×224 pixels). 2048 features are then extracted from these tiles with a convolutional neural network, pretrained using the self-supervised learning algorithm MoCo v2, described in previous work by Dehaene et al.^22^ Those 2048 features per tile are then fed into the tile-scoring and prediction step. Whenever multiple slides from the same patient are available, the model is applied independently to every slide and the predictions are averaged to obtain a patient-level risk score.

### Tile interpretability

The contributions of individual tiles to the final model prediction were computed using Shapley values.^23^^,^^24^ Those are naturally suited to our histology pipeline and were adapted as follows. A slide can be viewed as a set of N tiles, and we consider every possible way of subsampling those. For each subsampling, we obtain a subset of n tiles (n varying from 1 to N), and we compute the prediction of the model for this subset. In the framework of Shapley values, the contribution of a given tile i is defined as the average difference between the predictions obtained from subsets containing i and those obtained from subsets that do not contain i. This exact formula is intractable, since there are 2N possible subsets, with N as large as 50 000. In practice, we sample a maximum of 10 000 random subsets to obtain a robust estimate. 400 tiles (200 from the resection cohort and 200 from the transplant cohort) predictive of high-risk and low-risk were extracted and reviewed blindly by an expert hepatobiliary pathologist (DR) to assess for the presence of specific histologic features in tumor or non-tumoral tissue. The qualitative variables of high predictive value were compared using *Z*-tests of proportions, and the Holm-Sidak procedure was used to correct for multiple testing.

### Statistical analysis

Survival analyses were performed using univariate Cox proportional hazard models implemented in the lifelines package of Python. Log-rank tests were used to compare survival distributions between stratification subgroups. We used Harrel’s concordance index (C-index) as a metric for assessing the predictive performance of our deep learning and clinical models. The C-index evaluates whether the ranking of the model’s predictions is consistent with the ranking of the survival times of patients. Because of censored data, it is possible to rank 2 patients i and j with survival times ti and tj only if:

- ti>tj and patient j has had an event (death from disease or recurrence in our case).
- ti<tj and patient i has had an event.

Such a pair of patients is called “admissible,” and this pair is considered correctly classified by the model (“concordant”) if their risk score ri and rj satisfy:

- ri>rj if ti<tj (the risk should be higher for patients with a shorter survival).
- ri<rj if ti>tj.

The C-index is defined through the following formula:

C-index=number of concordant pairs / number of admissible pairs.

A C-index of 0.5 indicates a random performance, while a C-index of 1 indicates a perfect concordance between predictions and observations.

These results were validated on the discovery data set with the following cross-validation strategy: 5 stratified folds with 5 repeats. Folds were stratified based on censoring. C-indexes reported here are the average over the 25 folds. *p*-values were computed using bootstrap: patients were randomly sampled with replacement 10 000 times and the cross-validation average was computed for every sample, after which a Z-test was performed on the bootstrapped C-index difference. To assess the statistical significance of the stratification between high-risk and low-risk subgroups, we used a log-rank test as implemented in the python library lifelines.

## Results

### Patient characteristics

We identified 300 patients with HCC who underwent liver transplantation and 169 patients who underwent liver resection. Patient demographics are summarized in Table 1. Approximately, half (48%) of transplanted patients received pre-operative locoregional therapy, which primarily consisted of chemoembolization. None of the patients who underwent resection received pre-operative therapy. Nearly, half (44.8%) of patients in the transplant cohort had multiple tumors on pre-operative imaging, compared to 34% in the resection cohort. Pre-operative alpha-fetoprotein (AFP) levels were available for all 300 patients in the transplant cohort with a mean AFP of 63.1 ng/mL (median of 9.5 ng/mL). AFP levels were available in 160 patients in the resection cohort with a mean level of 7993.1 ng/mL (median of 16.4 ng/mL).

**Table 1:** Patient characteristics.

	Transplant cohort (*n*=300)	Resection cohort (*n*=169)
Mean age (years)	58.7 (35–76)	67.3 (39–88)
Gender Male Female Ratio	236 (79%) 64 (21%) 3.7	127 (75%) 41 (25%) 3.1
Race Caucasian Black Asian/Pacific Islander Hispanic Multiracial Not stated	248 (82.7%) 29 (9.7%) 8 (2.7%) 1 (0.3%) 7 (2.3%) 5 (1.7%)	124 (73.3%) 30 (1.8%) 6 (3.6%) 0 (0%) 1 (0.6%) 3 (1.8%)
Past medical history Alcohol use HCV infection HBV infection NAFLD/NASH Hemochromatosis Other	107 (36%) 182 (61%) 22 (7%) 62 (21%) 15 (5%) 27 (9%)	34 (20%) 69 (41%) 17 (10%) 24 (14%) 8 (4%) 6 (3%)
Pre-interventional imaging Multiple tumors Tumor(s) spanning multiple segments Tumor heterogeneity Cirrhosis	131 (44.8%) 127 (43.5%) 60 (20.5%) 286 (98.0%)	49 (34.0%) 67 (46.5%) 100 (69.4%) 47 (32.6%)
Preoperative therapy Locoregional therapy Chemotherapy/Immunotherapy Other	145 (48%) 1 (0.3%) 1 (0.3%)	0 0 0
Serum AFP level Mean preoperative AFP (ng/mL) Median preoperative AFP (ng/mL) Not available	63.1 (1.6-1816.7) 9.5 0	7993.1 (1.8-534397) 16.4 9
Tumor recurrence Yes No	46 (15%) 254 (85%)	64 (38%) 105 (62%)
Recurrence-free survival Mean RFS (months) Median RFS (months)	37.4 (3.2-173.7) 25.3	19 (2.2-72) 13
Outcome Alive Alive without evidence of HCC Alive with recurrent HCC Deceased Death from HCC Death from other causes	169 (56%) 166 (55%) 3 (1%) 131 (44%) 35 (12%) 96 (32%)	99 (59%) 51 (30%) 48 (28%) 70 (41%) 33 (20%) 37 (22%)

HCV, Hepatitis C virus; HBV, Hepatitis B virus; NAFLD, Non-alcoholic fatty liver disease; NASH, Non-alcoholic steatohepatitis; AFP, Alpha-fetoprotein; RFS, Recurrence-free survival.

Outcome data was available for all patients, with a median follow-up of 7 and 3 years for the transplant and resection cohorts, respectively. At the end of the follow-up period, 44% of transplanted patients and 41% of resected patients had died. Patient deaths were attributable to HCC recurrence at a rate of 27% in the transplant cohort and 47% in the resection cohort. More than half of patients in each cohort remained alive at the end of follow-up. While 96% of surviving post-transplant patients showed no evidence of recurrent HCC, only 52% of surviving patients in the resection cohort were HCC-free.

### Model development

In both the resection and transplant cohorts, we extracted a maximum of 20 000 randomly selected tiles (small image patches of 224×224 pixels) from each available WSI (out of a maximum of 65,042) and used a pretrained convolutional neural network to extract relevant features from each tile before training our models. To delineate the tumor area on H&E slides from the 2 Cleveland Clinic cohorts, we applied a tumor detection model that had been trained on previously annotated slides from the Mondor cohort.^21^ This tumor detection model is a ResNet50 neural network, trained to distinguish tiles containing tumors from those containing non-tumoral tissue. This detection model was trained on 240 000 tiles and tested on 60 000 tiles. Its performance was measured by the area under the receiver operating characteristic curve (ROC-AUC), which evaluates the capacity of the classifier to discriminate between 2 classes (ROC-AUC=0.5 for a random classifier, ROC-AUC=1 for a perfect classifier). The performance of the model on the test set was 0.98. This was then applied to unannotated slides from the Cleveland Clinic to separate tumor from non-tumoral areas.

**Fig. 1:**
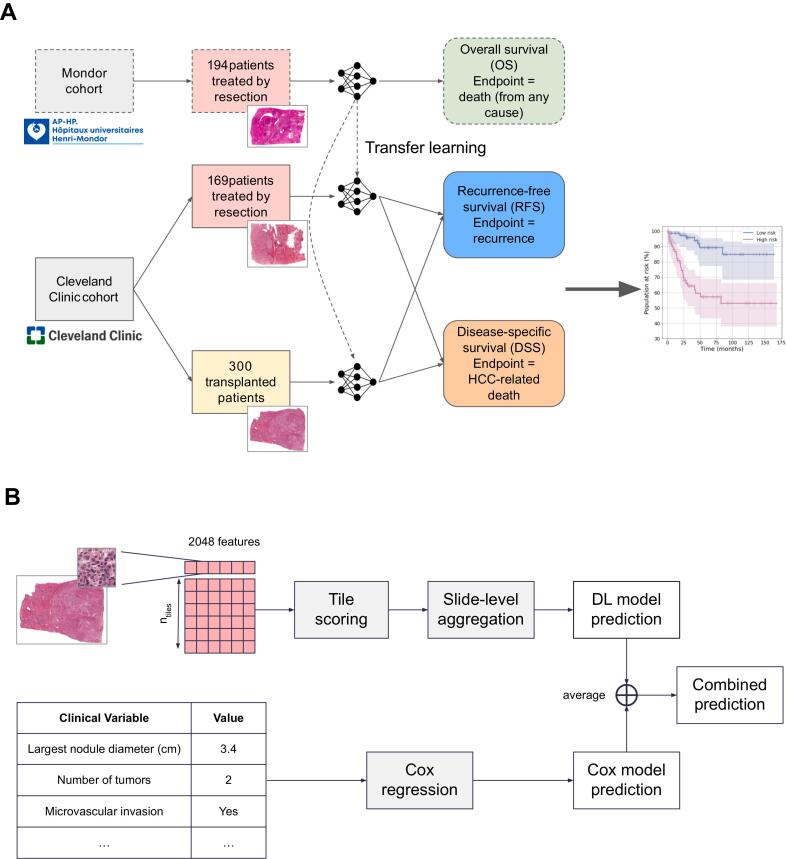
Flow chart showing methodology of the study (A) and schematic of model development (B). Models were first developed in a series of patients with HCC treated by surgical resection at Henri Mondor University Hospital (Créteil, France) to predict overall survival. Transfer learning was then used to predict recurrence-free survival and disease-specific survival in 2 cohorts of patients with HCC treated by surgical resection and liver transplant respectively at Cleveland Clinic (Cleveland, Ohio, USA).

The survival prediction model (Fig. 1A) was first trained with cross-validation for 20 epochs on a cohort of patients treated by resection at hospital Henri Mondor to predict overall survival, as described by Saillard et al.^21^ (C-index=0.78, std 0.07). Then, the model was fine-tuned for 10 epochs separately on each of the 2 Cleveland Clinic cohorts to predict either recurrence-free survival (RFS) or disease-specific survival (DSS). For both pretraining and fine-tuning, smooth C-index loss^23^ was used as a training objective.

**Fig. 2:**
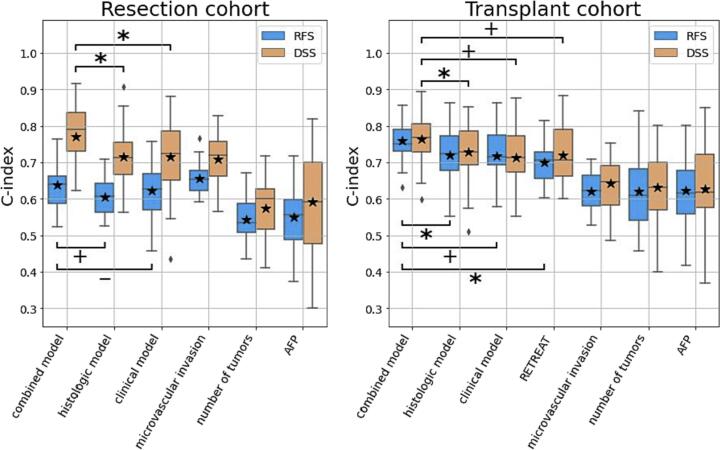
Predictive performance of the RFS and DSS models in resection (left) and transplant (right) cohorts, as measured by C-index. In both cohorts, the combined model outperforms both the separate clinical and histologic model. In the transplant cohort, the combined model also outperforms the RETREAT score. AFP=preoperative Alpha-Feto-Protein. *p*-values are as follows: *: <.05, +: <.1, −: >.1.

We assessed the discriminatory power of our deep learning model for predicting RFS or DSS by cross-validation. On the resection cohort, this deep learning model was predictive of both RFS (average C-index of 0.61) and DSS (average C-index of 0.72) (Fig. 2). To compare the performance of this model to a separate model considering only clinical variables, we performed univariate and multivariate analyses of the collected clinical data. The presence or absence of microvascular invasion was the most predictive variable overall, and the multivariate Cox regression of clinical data showed similar performance to the deep learning model in both RFS and DSS, with C-indexes of 0.62 and 0.72, respectively. However, the integration of the deep learning model with the clinical data outperformed both modalities alone. This was done by averaging the predictions of the deep learning model and the Cox regression of clinical data (Fig. 1B). This combined clinical and histologic model reached a C-index of 0.64 and 0.77 when predicting RFS and DSS, respectively, on the resection cohort.

In the transplant cohort, applying the separate histologic and clinical models, as well as the combined model, showed good performance on both target outcomes. The deep learning model reached an average C-index of 0.72 for RFS and 0.73 for DSS (Fig. 2). The multivariate Cox regression of clinical variables outperformed every univariate prediction for both RFS and DSS (average C-index of 0.72 and 0.71, respectively). However, the combined clinical and histologic model significantly outperformed both the multivariate clinical and deep learning histologic models independently, reaching a C-index of 0.76 for RFS and 0.77 for DSS. This combined model performed better on the subset of transplant patients who did not receive locoregional therapy (C-index of 0.83 and 0.87 for RFS and DSS, respectively) but still maintains good predictive performance even when locoregional therapy was administered prior to transplantation (C-index of 0.71 and 0.72 for RFS and DSS, respectively) (Supplementary Fig 1).

In order to compare our results with an independent prognostic system, we also calculated the RETREAT score^18^ for all cases in our transplant cohort. Since the RETREAT system uses recurrence as an endpoint rather than survival, only RFS was considered. The RETREAT score reached a C-index of 0.69 to predict RFS, while our combined model showed significantly better predictive performance (C-index of 0.76, *p*=.03).

**Fig. 3:**
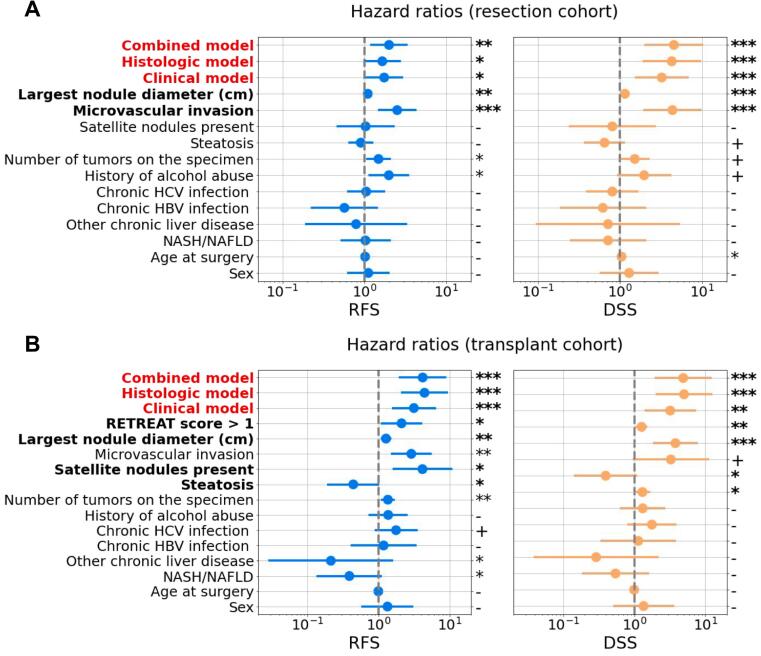
Hazard ratio and 95% confidence intervals for RFS and DFS predictions by the 3 models and independent clinical variables, for resections (A) and transplants (B) respectively. Model predictions were converted into binary scores (high-risk or low-risk) using the median as a threshold. Variables significant for both outcomes are indicated in bold font. *p-*values are as follows: ***:<.001, **: <.01, *: <.05, +: <.1, −: >.1.

**Fig. 4:**
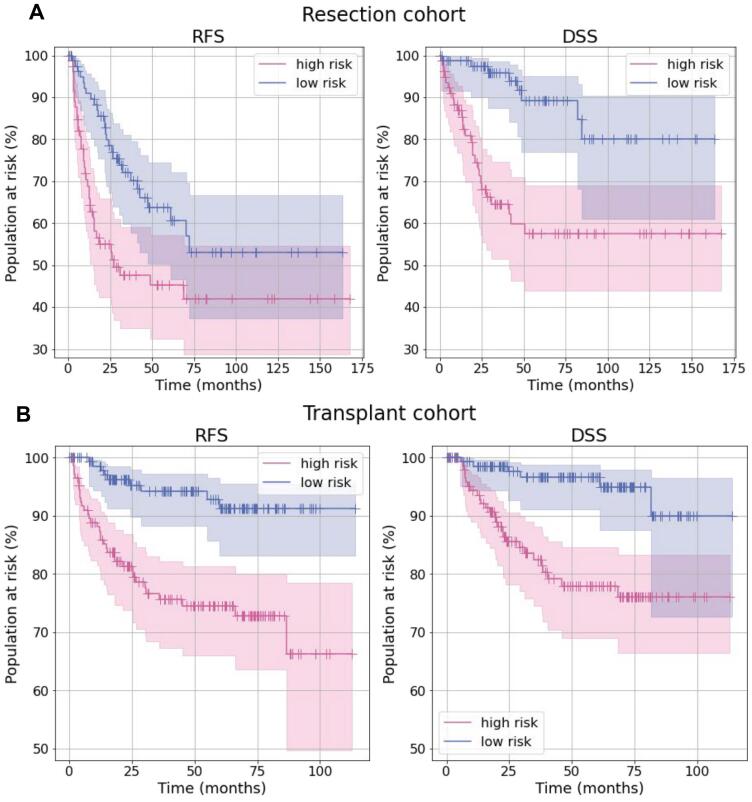
Kaplan–Meier curves of high-risk and low-risk groups stratified using the combined model on the resection cohort for RFS (A, left, log-rank *p*-value=.0090) and DSS (A, right, log-rank *p*-value=9.3×10^−6^), and on the transplant cohort for RFS (B, left, log-rank *p*-value=3.2×10^−5^) and DSS (B, right, log-rank *p*-value=1.4×10^−4^).

As the output of our model is a continuous score, we stratified the population into high-risk and low-risk groups based on the score assigned to each patient. The median score of each model was used as the threshold for stratifying patients into these 2 subgroups. The clinical, histologic, and combined models were all able to separate the population groups accurately. In the resection cohort, the combined model stratified the population with hazard ratios (HR) of 2.00 (*p*-value=.0059) to predict RFS and 5.55 (*p*-value=4.10-6) to predict DSS (Figs. 3A, 4A). In the transplant cohort, the HR was 4.17 (*p*-value=2.10-5) to predict RFS and 4.73 (*p*-value=8.10-5) to predict DSS (Figs. 3B, 4B). Of note, this combined model was also able to accurately stratify the subgroups of transplanted patients who did or did not receive locoregional therapy (Supplementary Fig 2).

**Fig. 5:**
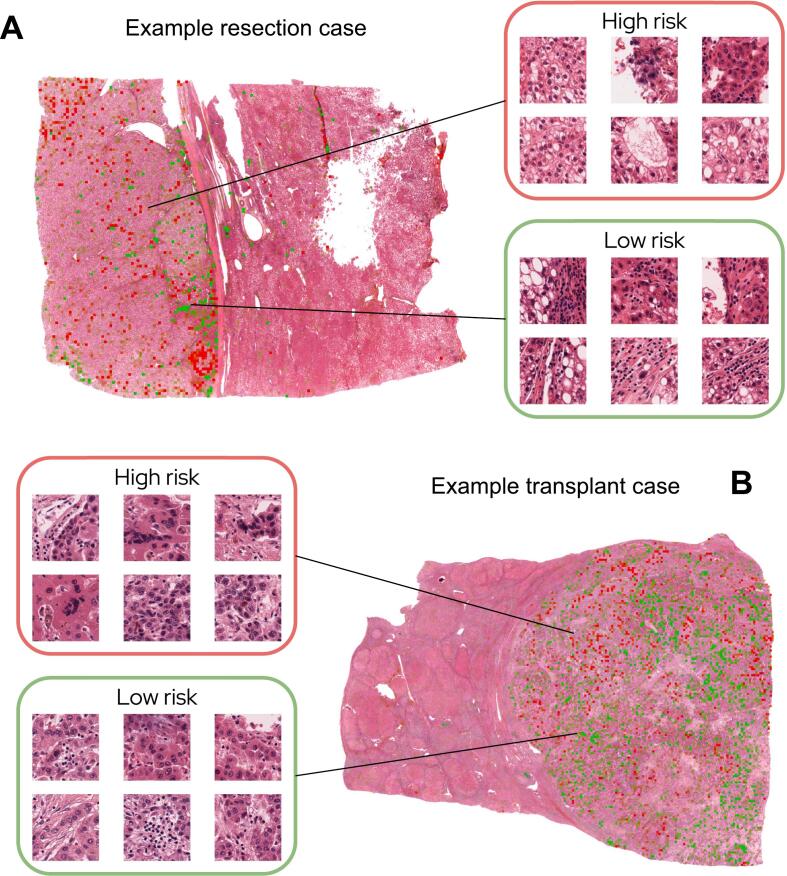
A heatmap visualization is shown from the resection cohort (A) with examples of high-risk and low-risk tiles. The bottom set of images contains an example heatmap from the transplant cohort (B) with corresponding high-risk and low-risk tiles.

In order to better understand the deep learning model’s risk assessment criteria, we extracted the 400 most predictive tiles (100 tiles associated with high-risk scores and 100 associated with low-risk scores from each cohort) (Fig. 5). These tiles were blindly reviewed by an expert hepatobiliary pathologist (DR), who documented the presence or absence of fifteen histologic features in tumor areas and five features in non-tumoral areas (Table 2, Table 3). The histologic parameters identified by the deep learning model as most predictive of HCC recurrence or death were the presence of macronucleoli (*P=*1.4×10^−3^), a nuclear-to-cytoplasmic ratio greater than 50% (*P=*1.1×10^−2^), significant nuclear pleomorphism (*P*=9.4×10^−4^), and necrosis (*P=*2.3×10^−5^). The model also determined that the presence of lymphocytic inflammation was a low-risk feature, both within areas of tumor (*P=*1.7×10^−9^) and in non-tumoral tissue (*P*=1.2×10^−4^).

**Table 2:** Histologic features associated with high or low risk (tumor tiles).

	Total	High risk	Low risk	Significance
Lymphocytic inflammation	60 (22%)	8 (5%)	52 (47%)	***
Macronucleoli	71 (26%)	61 (37%)	10 (9%)	**
Nuclear pleomorphism	43 (16%)	38 (23%)	5 (5%)	**
Nuclear-to-cytoplasmic ratio >50%	50 (18%)	43 (26%)	7 (6%)	*
Cytoplasmic amphophilia	56 (20%)	41 (25%)	15 (14%)	–
Nuclear hyperchromasia	46 (17%)	34 (20%)	12 (11%)	–
Steatosis	27 (10%)	9 (5%)	18 (16%)	–
Blood/hemorrhage	18 (6%)	12 (7%)	6 (5%)	–
Fibrosis	16 (6%)	14 (8%)	2 (2%)	–
Clear cytoplasm	11 (4%)	8 (5%)	3 (3%)	–
Bile	10 (4%)	9 (5%)	1 (1%)	–
Mitoses	2 (1%)	1 (1%)	1 (1%)	–
Necrosis	2 (1%)	2 (1%)	0 (0%)	–
Vascular invasion	2 (1%)	2 (1%)	0 (0%)	–
Solid architecture	1 (0.4%)	1 (1%)	0 (0%)	–
Total	277	167	110

**Table 3:** Histologic features associated with high or low risk (non-tumor tiles).

	Total	High risk	Low risk	Significance
Lymphocytic inflammation	69 (57%)	1 (3%)	68 (76%)	***
Necrosis	11 (9%)	11 (34%)	0 (0%)	***
Blood/hemorrhage	16 (13%)	9 (28%)	7 (8%)	-
Fibrosis	25 (21%)	7 (22%)	18 (20%)	-
Steatosis	4 (3%)	0 (0%)	4 (4%)	-
Total	121	32	89	

Despite the preprocessing, some artifacts (like tissue folding) remained present in our dataset. To assess whether these had any impact on the model’s predictions, the presence of artifacts was also annotated by the pathologist. Of the 400 tiles analyzed, 21 had artifacts, and they were equally present in high-risk and low-risk tiles (12 and 9 tiles respectively, *p*-value non-significant), indicating that tissue artifacts had no impact on the model’s predictions.

**Fig. 6:**
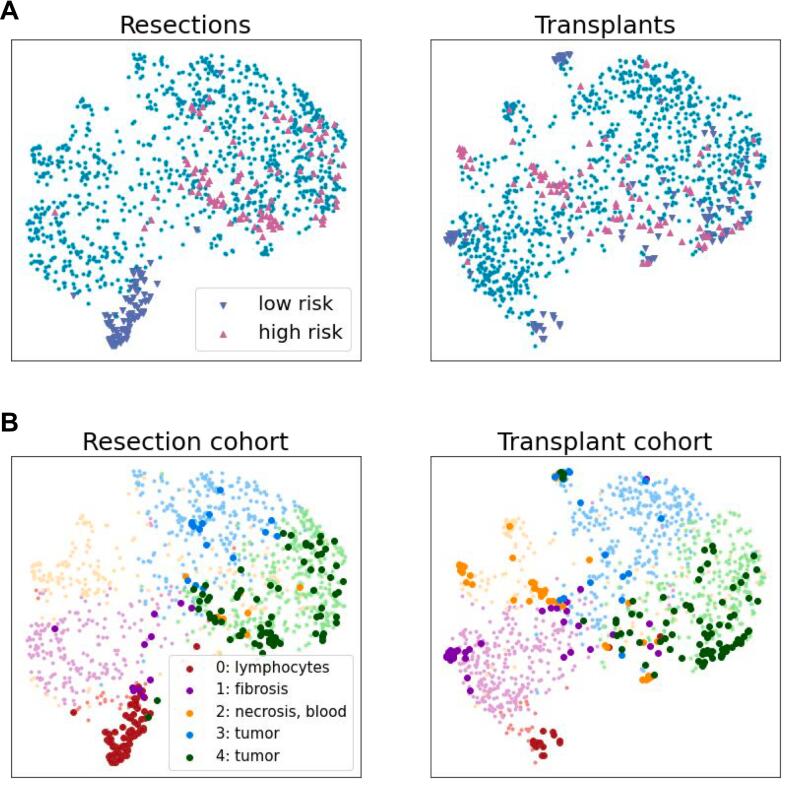
UMap visualization of 1200 tiles per cohort, including the 200 high-risk and low-risk tiles that were reviewed, and 1000 intermediate tiles. High-risk and low-risk tiles are visualized on both cohorts (A). KMeans clustering with 5 clusters was applied to the selected tiles (B). Cluster 0 is characterized by the presence of lymphocytes, cluster 1 by fibrosis, and cluster 2 by necrosis. Clusters 3 and 4 contain the majority of tumor tiles, with cluster 4 composed mainly of high-risk patterns such as macronucleoli, nuclear pleomorphism, and increased nuclear-to-cytoplasmic ratio. High-risk and low-risk tiles are indicated by larger circles and a darker shade.

Finally, in each cohort, we applied the UMAP (Uniform Manifold Approximation and Projection) algorithm to visualize the similarities and differences between high- and low-risk tiles (Fig. 6, Supplementary Fig 3). We used the previously characterized set of 400 tiles extracted from both cohorts. This set was augmented with a random selection of tiles (1000 per cohort) covering the entire distribution of predicted risks. UMAP was fitted on the features of the resulting set of 2400 tiles to obtain a common representation for resections and transplants. A clustering algorithm (KMeans) was then applied to divide tiles into 5 clusters, corresponding to various histologic features. In resections, low-risk tiles are mostly located in cluster 0, which is characterized by a significant lymphocytic infiltrate, while low-risk tiles in the transplant cohort are much more morphologically diverse. Most tumor tiles in both cohorts belong to clusters 3 and 4, with the latter containing the high-risk histologic features of macronucleoli, nuclear pleomorphism, and high nuclear-to-cytoplasmic ratio. Some high-risk tiles from the transplant cohort are also located in cluster 1, consisting mainly of necrotic areas.

## Discussion

In this study, we built upon a previously developed deep learning model derived from HCC resection specimens.^21^ We expanded this model with the addition of clinical data and applied it to new cohorts, which included both HCC resections and transplant specimens. This combined model showed excellent predictive power, significantly improving upon the RETREAT score when assessing tumor recurrence in our transplant cohort.

Our deep learning model utilized a weakly supervised approach, and the specific features present in high-risk and low-risk tiles were blindly characterized by an expert hepatobiliary pathologist. Given that high-risk histologic features of HCC have been previously described in numerous other studies,^17^^,^^25^, ^26^, ^27^, ^28^, ^29^ this step allows us to compare the model’s criteria with documented high-risk histologic parameters as a quality check. Our model considered the presence of a high nuclear-to-cytoplasmic ratio, nuclear pleomorphism, macronucleoli, and necrosis as high-risk features, correlating with tumor recurrence and poor survival. These results are concurrent with HCC literature, as these same histologic features have been described in numerous prior studies, thereby reinforcing our confidence in the deep learning model.^17^^,^^25^, ^26^, ^27^, ^28^, ^29^ Our model also considered the presence of lymphocytic inflammation to be a low-risk feature. Indeed, tumor-infiltrating lymphocytes (TIL) represent a well-documented histologic pattern associated with favorable prognosis in many cancer types.^30^, ^31^, ^32^ While studies describing the prognosis of TILs within HCC are scarce, the lymphocyte-rich subtype of HCC is reported to have a more favorable prognosis than other morphologies.^33^, ^34^, ^35^ Overall, the concordance between our histologic model’s risk evaluation and the overall body of pathology literature on this topic helps to confirm validity of the model’s output.

Methods of determining pretransplant eligibility and posttransplant prognosis have evolved over the past few decades. Traditional eligibility systems involve only the radiographic measurement of tumor extent,^15^^,^^36^ while more recent pretransplant eligibility systems have begun to incorporate an interdisciplinary approach that includes both lab testing and biopsy results, such as the extended Toronto criteria.^37^ Models that predict post-transplant outcome have similarly begun to consider criteria spanning multiple disciplines. While well-documented histologic features such as microvascular invasion and tumor differentiation remain prognostically significant, outcome models using only clinical parameters have also been developed,^16^^,^^38^ and the recent RETREAT system combines both clinical and histologic variables to improve the prediction of HCC recurrence following transplantation.^18^

The adoption of AI in this field has taken a similar incremental approach,^19^^,^^20^^,^^39^, ^40^, ^41^ with some predictive algorithms built solely on clinical variables and other deep learning models derived from whole-slide images alone. In this study, we show that the combination of both clinical and histologic inputs improved the predictive performance compared to either modality alone. We also demonstrated the expandability of this deep learning model, as the initial series was trained on HCC resections alone, yet showed excellent performance when applied to transplant patients in this study.

Finally, the addition of clinical data significantly increased the model’s predictive performance, demonstrating that AI modeling can be improved not only through the addition of more cases, but by incorporating clinical variables and reproducing the evolving interdisciplinary approaches seen in non-AI prognostic models. Future integration of radiographic imaging and genomic data may further optimize the efficacy of this model and help realize a more personalized approach to HCC surveillance after surgery.

## Funding

The authors received no specific funding for this work.

## Declaration of Competing Interest

The authors do not have any conflicts of interest.

## Data Availability

De-identified histopathology images are available from the corresponding author with a material transfer agreement.

## Appendix A Supplementary data

Supplementary material 1 The performance of outcome prediction for various models (combined, histologic, clinical, RETREAT) and clinical variables (microvascular invasion, tumor number, AFP level) within the transplant cohort are shown, as measured by C-index and stratified by those who received locoregional therapy (right) and those who did not (left). Supplementary material 1

Supplementary material 2 Kaplan-Meier curves are shown of high-risk and low-risk patient subgroups (identified by the combined model) among the transplant cohort, stratified by those who did not receive locoregional therapy (A, log-rank *p*-value=.03 for RFS and log-rank *p*-value=.01 for DSS), and those who did (B, log-rank *p*-value=.04 for RFS and log-rank *p*-value=2.9x10^-5^ for DSS). Supplementary material 2

Supplementary material 3 UMap visualization of 1200 tiles from the transplant cohort, which includes the 100 high-risk and 100 low-risk tiles that were reviewed, plus 1000 additional intermediate tiles. High- and low-risk tiles are visualized on patients who either did or did not receive locoregional therapy (A). The five clusters defined in Fig. 6 are visualized within each subgroup (B). High-risk and low-risk tiles are indicated by larger circles and a darker shade. Supplementary material 3
