# Supplementary figures and images for: Combining a deep learning model with clinical data better predicts hepatocellular carcinoma behavior following surgery
Source: J Pathol Inform. 2023 Dec 29;15:100360. doi: 10.1016/j.jpi.2023.100360 (PMC10825615; doi:10.1016/j.jpi.2023.100360)

# No locoregional therapy

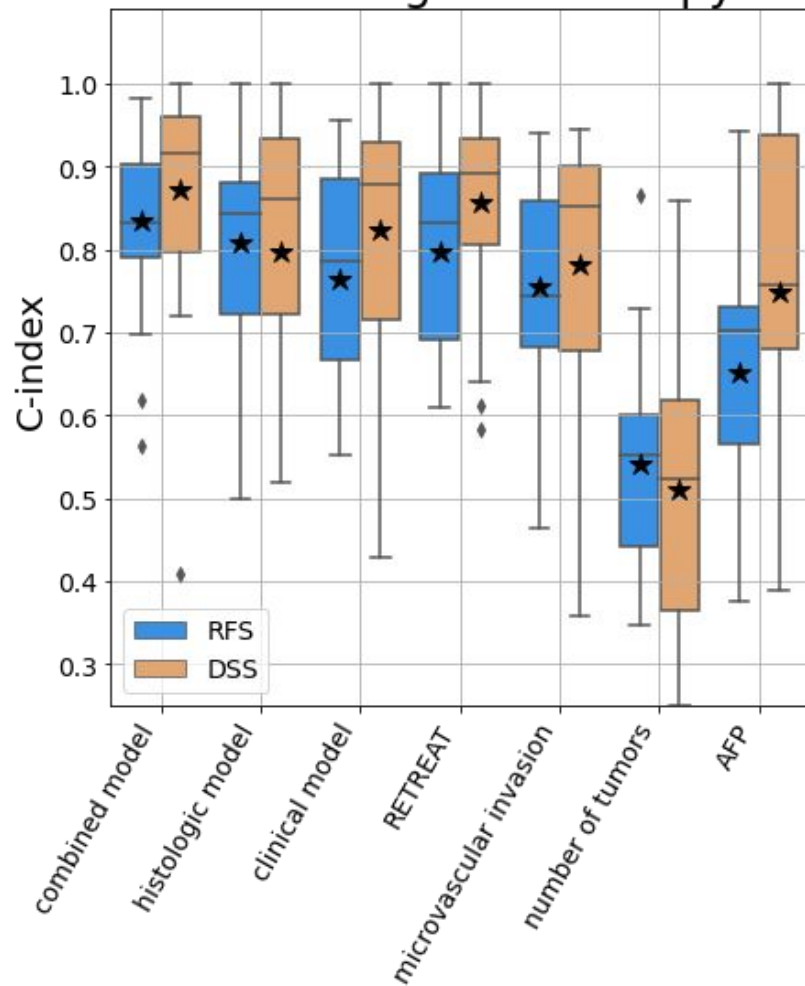

# Received locoregional therapy

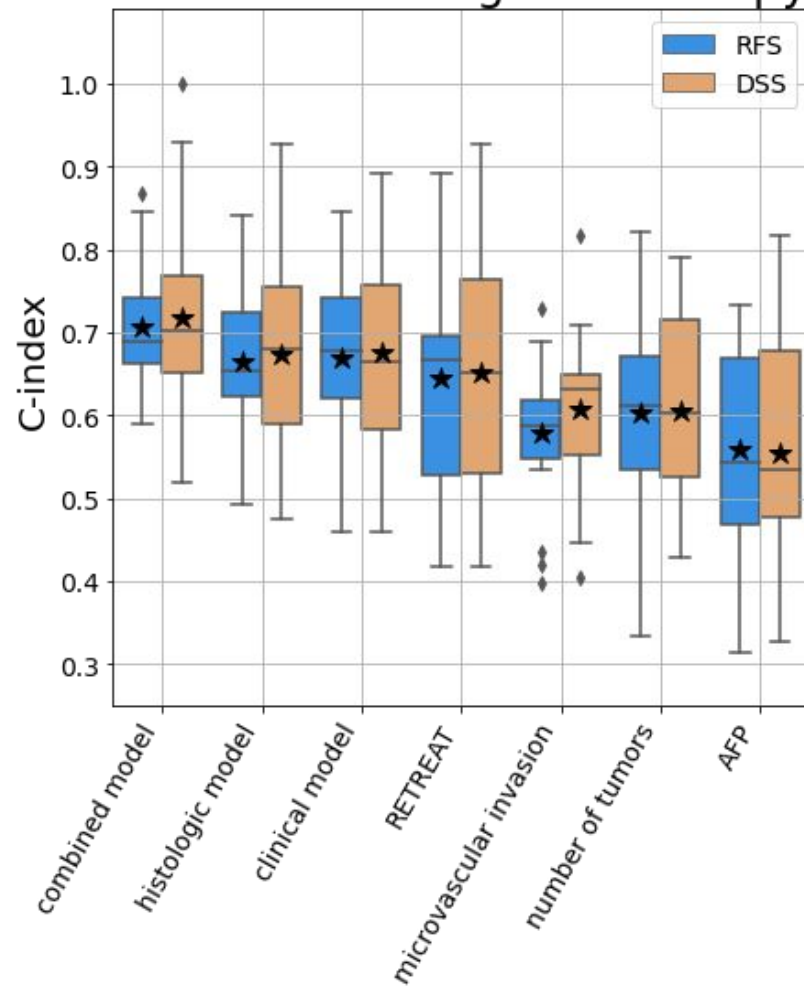

Supplement: Supplementary material 1 — The performance of outcome prediction for various models (combined, histologic, clinical, RETREAT) and clinical variables (microvascular invasion, tumor number, AFP level) within the transplant cohort are shown, as measured by C-index and stratified by those who received locoregional therapy (right) and those who did not (left). [file mmc1.pdf]

**A**

No locoregional therapy

RFS

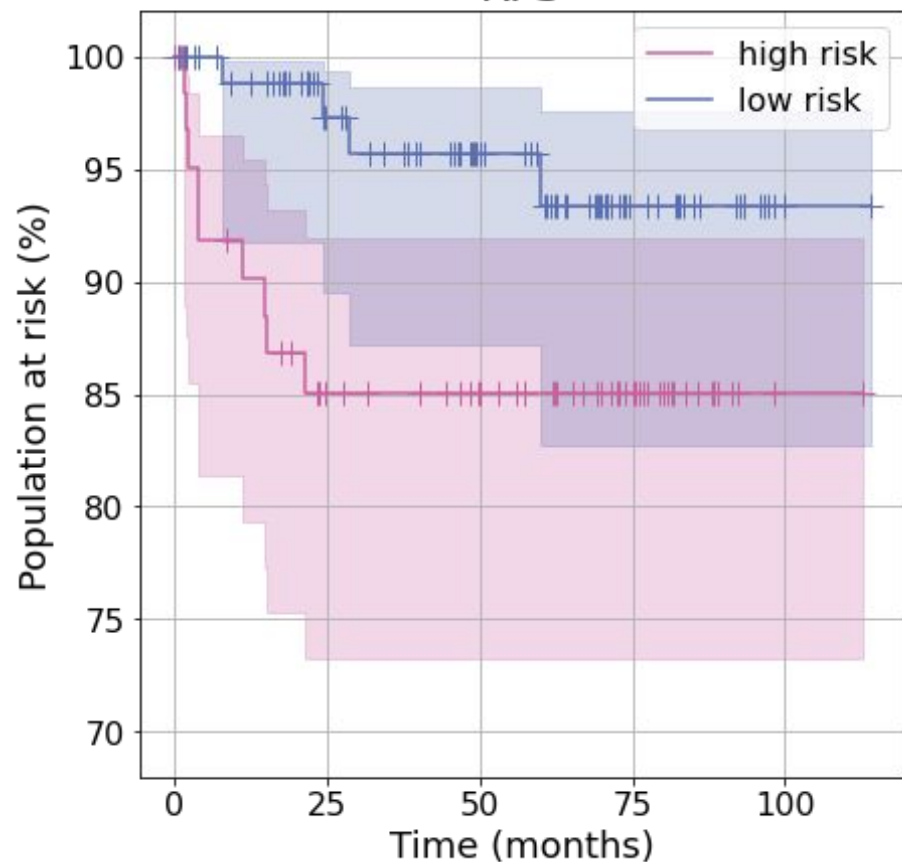

DSS

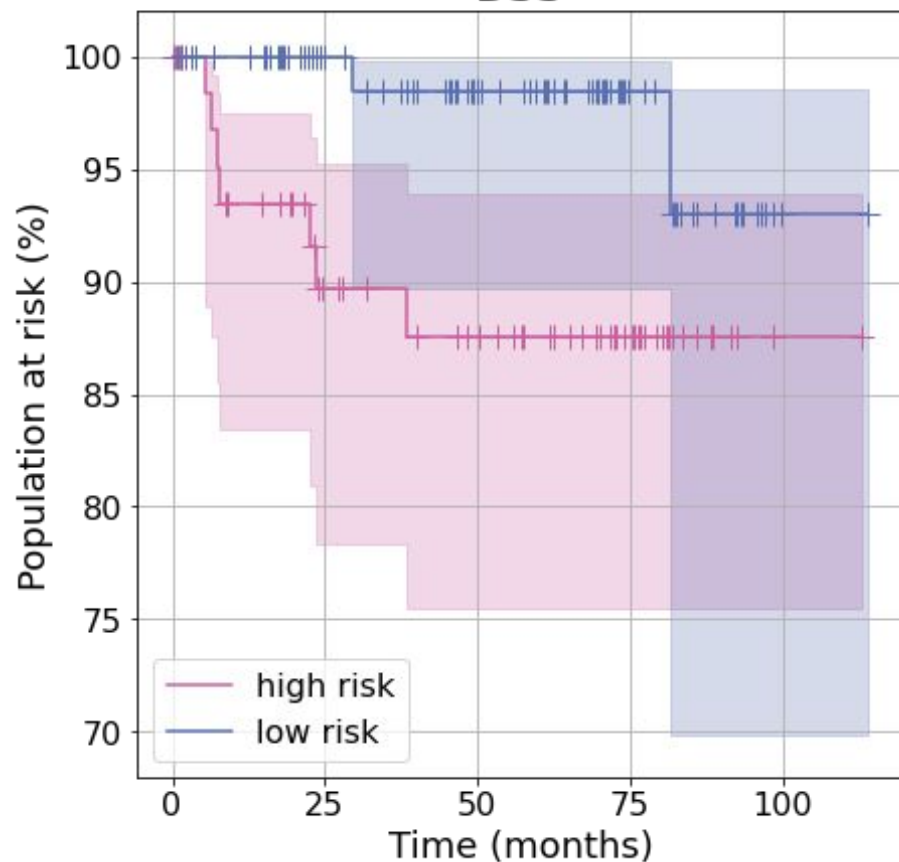**B**

Received locoregional therapy

RFS

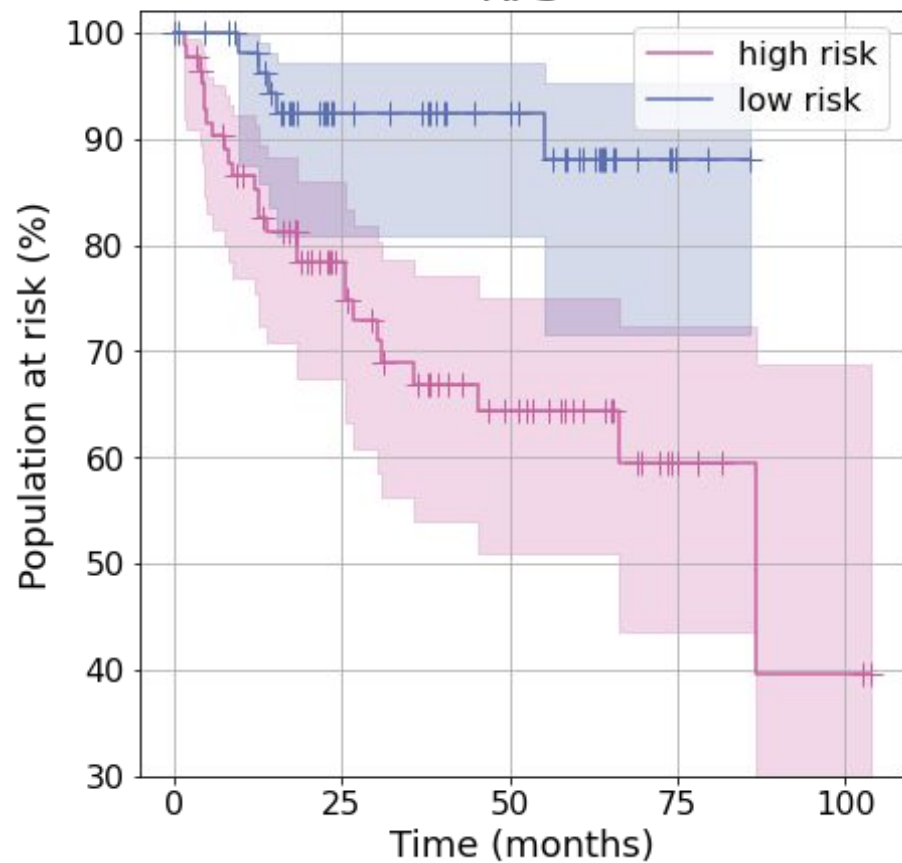

DSS

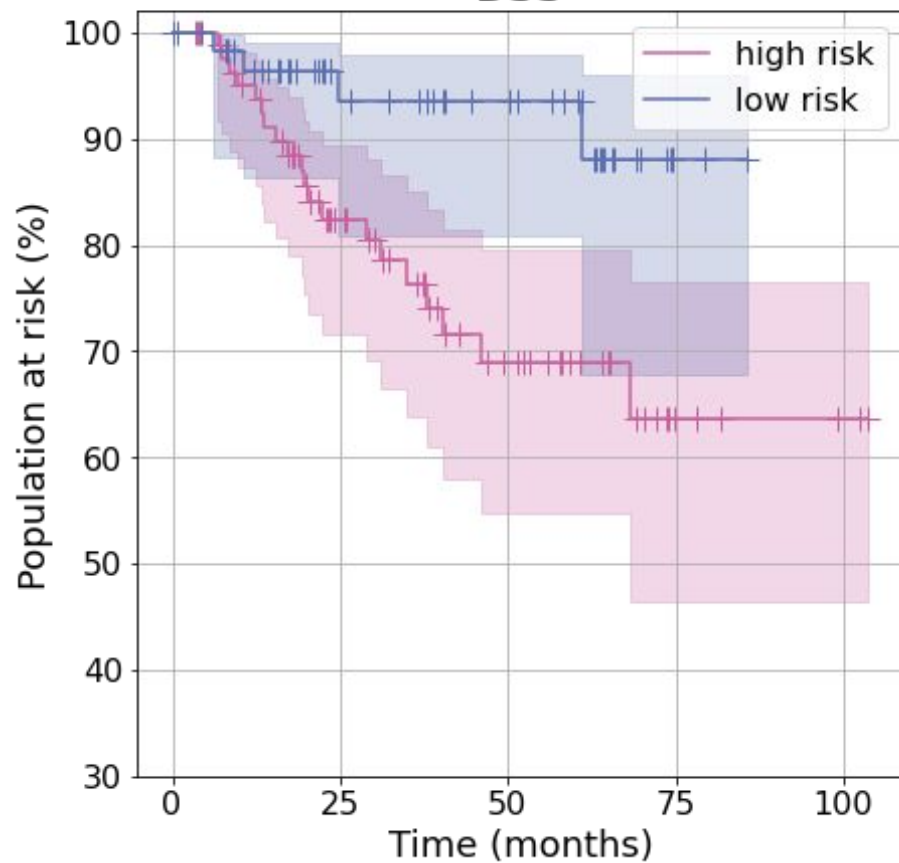

Supplement: Supplementary material 2 — Kaplan-Meier curves are shown of high-risk and low-risk patient subgroups (identified by the combined model) among the transplant cohort, stratified by those who did not receive locoregional therapy (A, log-rank p-value=.03 for RFS and log-rank p-value=.01 for DSS), and those who did (B, log-rank p-value=.04 for RFS and log-rank p-value=2.9x10^-5^ for DSS). [file mmc2.pdf]
