# Supplementary material for: Combining a deep learning model with clinical data better predicts hepatocellular carcinoma behavior following surgery
Source: J Pathol Inform. 2023 Dec 29;15:100360. doi: 10.1016/j.jpi.2023.100360 (PMC10825615; doi:10.1016/j.jpi.2023.100360)
Supplement: Supplementary material 3 — UMap visualization of 1200 tiles from the transplant cohort, which includes the 100 high-risk and 100 low-risk tiles that were reviewed, plus 1000 additional intermediate tiles. High- and low-risk tiles are visualized on patients who either did or did not receive locoregional therapy (A). The five clusters defined in Fig. 6 are visualized within each subgroup (B). High-risk and low-risk tiles are indicated by larger circles and a darker shade. [file mmc3.pdf]

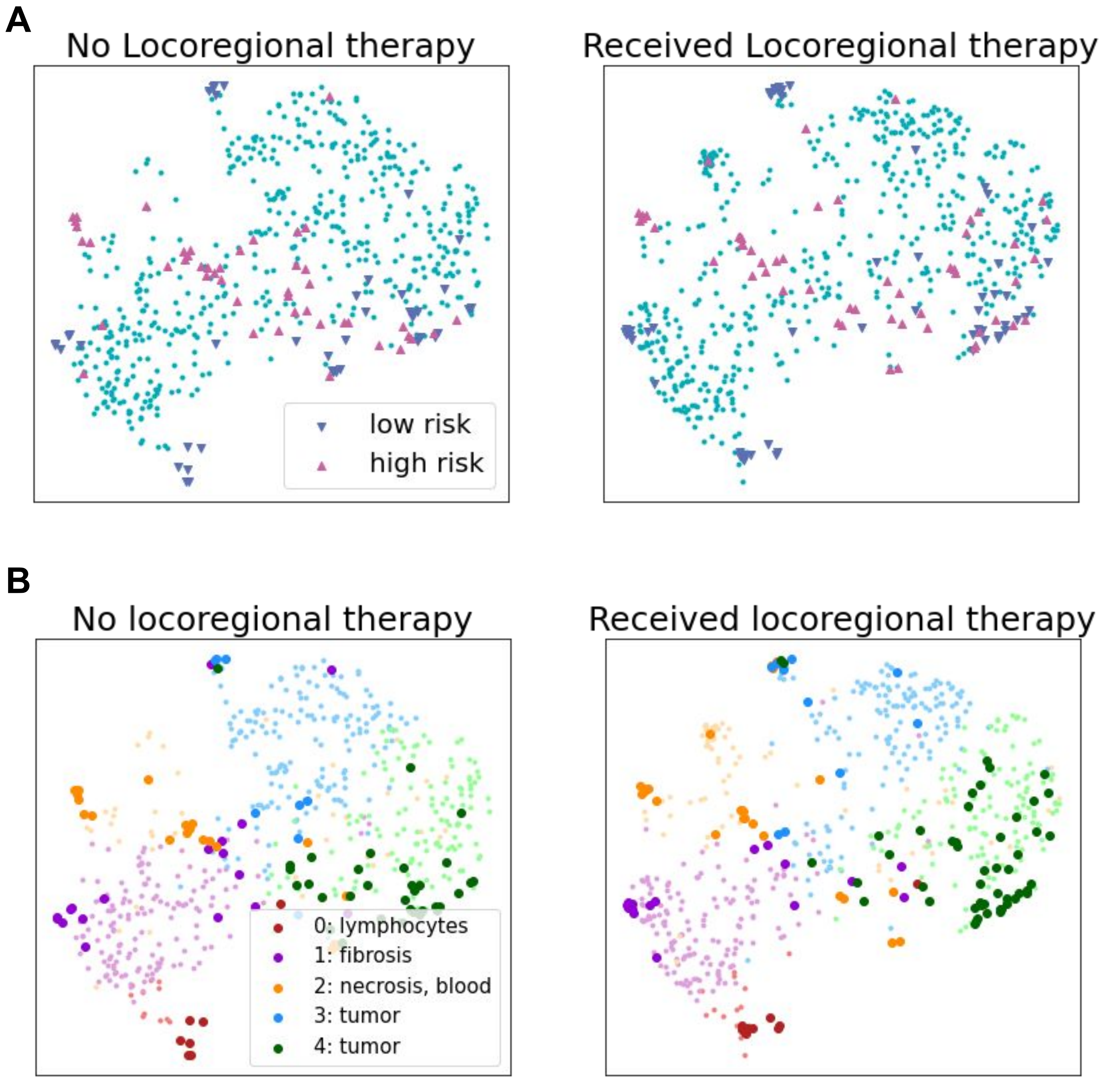
A
No Locoregional therapy
Received Locoregional therapy
low risk
high risk
B
No locoregional therapy
Received locoregional therapy
0: lymphocytes
1: fibrosis
2: necrosis, blood
3: tumor
4: tumor
